# Characterization of Solid Renal Masses using 64-Slice Multidetector CT Scanner

**DOI:** 10.1100/tsw.2009.65

**Published:** 2009-06-12

**Authors:** Saleh S. El-Esawy, Mohamed E. Abou El-Ghar, Ghada M. Gaballa, Saly A. Zahra

**Affiliations:** Mansoura University, Egypt

**Keywords:** renal, mass characterization, CT

## Abstract

The purpose of our study was to assess the role of a 64-slice multidetector CT (MDCT) scanner in the characterization of different solid renal masses, using a simplified approach to correct the postenhancement attenuation values. The study included 96 consecutive adults (58 men, 38 women) with renal masses; 93 with unilateral and three with bilateral masses. All of our patients underwent multiphasic CT study including pre- and postcontrast corticomedullary (CM) and nephrographic phases. We analyzed the images and corrected the postcontrast attenuation values at the CM phase. The postbiopsy or -surgical data were used as reference standard. There were 53 masses at the right kidney, 40 at the left kidney, and three bilateral. The final diagnosis of the 96 solid parenchymal masses were 28 clear-type renal cell carcinoma (RCC), 22 papillary-type RCC, 21 chromophobe-type RCC, six XP 11.2 chromosomal translocation–type RCC, 15 angiomyolipoma (AML), and seven oncocytoma. All the AML had fat, with attenuation values less than -40 HU at the nonenhanced scan. There is no difference in the precontrast attenuation values for the different types other than AML. At the postcontrast CM phase after the correction of the attenuation values, the clear cell type could be separated easily, with attenuation values >20 with specificity, sensitivity, and overall accuracy of 92, 84, and 93%, respectively. The 64-slice MDCT scanner with application of enhancement values correction allows diagnosis of clear cell carcinoma. Also, AML could be identified easily with fat inside at the precontrast scan.

## INTRODUCTION

The great majority of renal masses are found incidentally as a result of the wide use of computed tomography (CT), ultrasonography (US), and magnetic resonance (MR) imaging. Fortunately, most of these are simple renal cysts that can be easily diagnosed and do not require treatment. However, solid and complex cystic renal masses are also discovered, many of which are clearly malignant and need to be surgically removed, while others may not require surgical intervention[1].

There was a time when renal cancer was just a solid enhancing mass in the kidney that required no further description and was removed with radical nephrectomy. Since then, advances in our understanding and the treatment of renal cancer have occurred that bring into question the validity of several aspects of this practice paradigm. One manifestation of the evolution of our knowledge of renal cancer is the discovery of an increasingly complex array of tumor subtypes; these tumor subtypes range from the common to almost unheard of[2].

Multidetector computed tomography (MDCT) is the latest breakthrough in CT technology. Thin sections can now be acquired on a routine basis in a single-breath hold with 3D-isotropic reconstruction. This results in improving the lesion detection of benign as well as malignant abdominal tumors. The ability to scan through the entire abdomen in seconds allows multiphasic acquisition; therefore, precise timing and optimized contrast is of great importance[3].

Some histological subtypes of renal cell carcinoma (RCC) have unique imaging findings, which may permit prediction of histology with its attendant implication for management and prognosis. Also, the tumor response to molecular therapeutics may be vastly different than the response to standard cytoreductive therapy[4].

Accurate histological and imaging characterization of RCC is very important from prognostic and management perspectives[5,6,7]. It is well established that clear cell RCC is associated with a less favorable prognosis compared with papillary and chromophobe carcinoma[5,7]. It is also well known that collecting duct carcinomas and renal medullary carcinomas are associated with aggressive clinical behavior and poor prognosis[6,8,9,10]. Precise classification of RCC also allows the institution of tailored treatment protocols[2].

The aim of our study was to assess the role of a 64-slice MDCT scanner in the characterization of different renal mass subtypes, using a simplified approach to correct the postenhancement attenuation values.

## MATERIAL AND METHODS

Our institutional ethical committee reviewed and approved the study protocol**.** In our prospective study, we included 96 patients with renal masses from March 2007 until September 2008 (58 men and 38 women). Their mean age was 54 ± 13.5 years (range: 14–79 years). There were 53 masses on the right, 40 on the left, and three were bilateral. All of our patients had a solitary tumor except three with bilateral angiomyolipomas (AML); the total number of masses was 99. There were 77 patients with RCC, seven with oncocytoma, and 12 with 15 AML. All of our patients had serum creatinine <1.8 mg/dl; the mean 1.4 mg/dl (range: 1.1–1.8 mg/dl).

All of our patients underwent multiphasic helical CT study and informed consent was obtained from all patients. Pregnancy tests were given to female patients of productive age.

### Protocol of CT

The study was done on a 64-multislice helical CT scanner (Brilliance, Philips, The Netherlands) with a standard uniform protocol for all of our patients. We injected 120 ml of contrast material (Ultravist 300 [iopromide], Schering, Berlin) at a flow rate of 5 ml/sec using an automatic injector. The contrast was injected via the anticubital vein via a 19-gauge cannula.

The multiphasic CT study included a precontrast scan of the upper abdomen from the diaphragm to the iliac crest, corticomedullary (CM) phase after 25 sec from injection of contrast for the kidney, then after 10-sec delay after CM phase, we obtained nephrographic phase from the diaphragm to iliac crest. The slice section for noncontrast-postcontrast phase was 5 mm with an overlap of 2.5 mm, and for CM and nephrographic phases was 2–5 mm with 1.25 mm overlap.

All examinations were reviewed on PACS system (Magic View, GE, Milwaukee, WI) by two experienced radiologists and the cases were diagnosed by consensus.

For measurement of attenuation value, we got a region of interest (ROI) at each phase. The ROI was applied to the image with a large solid component and it was applied to include most of the mass excluding the margin, the cystic necrosis, or calcific foci. In cases of AML, we included the fatty areas inside the ROI.

To eliminate the influence of intrinsic factors on the measured attenuation values, we divided the attenuation value of the mass at the CM phase by the attenuation of the aorta at CM phase; then we multiplied it by 100 to obtain the corrected attenuation. Then we subtracted the attenuation value of the mass at nonenhanced scan from the corrected attenuation value at the CM phase to obtain the relative enhancement of the mass.

$$\text{Corrected attenuation} = \frac{\text{Attenuation value of the mass at CM phase}}{\text{Attenuation value of the aorta at CM phase}} \times 100$$

Relative enhancement of the mass = the corrected attenuation at CM phase - the attenuation value at precontrast scan.

The 12 patients with AML underwent percutaneous fine-needle biopsy to confirm the diagnosis, 24 patients underwent partial nephrectomy for small localized masses, and the remaining 60 patients underwent radical nephrectomy. We used the histopathology as the reference standard.

### Statistical Analysis

We identified if the mass had a positive or negative relative enhancement after correction of enhancement, and we use paired t-test with $p$ value $< 0.05$ considered statistically significant. We also compared the size of the different masses.

## RESULTS

The final diagnosis of our patients was 28 clear cell RCC, 22 papillary RCC, 21 chromophobe RCC, six XP 11.2 chromosomal translocation RCC, 15 AML, and seven oncocytoma.

Among the cases of AML (Fig. 1), all could be diagnosed easily by the presence of fat inside. The fat could be identified easily at unenhanced scan with attenuation values less than –40 HU. For the remaining 84 cases, we calculated the mean attenuation value at the precontrast and CM phase. At the postenhanced scan, the mean relative enhancement for the papillary RCC (Fig. 2) was –4.9 ± 10.1 (range: –15 to 8 HU), for clear cell carcinoma (Fig. 3) 30.7 ± 11.8 (range: 7–52 HU), for the chromophobe RCC (Fig. 4) 1.8 ± 12.4 (range: –19 to 18 HU), for XP 11.2 chromosomal translocation RCC type 17.4 ± 16.2 (range: 0–45 HU), and for oncocytoma 14 ± 17.3 HU (range: 13–35 HU).

At precontrast scan, the mean attenuation value of papillary RCC was 32.6 ± 11 (range: 21–70 HU), for clear cell RCC 33.8 ± 8.8 (range: 22–57 HU), for chromophobe RCC 31.9 ± 5.6 (range: 22–44 HU), for XP 11.2 chromosomal translocation RCC 27.1 ± 3.4 (range: 22–32 HU), and for oncocytoma 29.6 ± 9.1 (range: 17–45 HU) (Table 1).

The mean size of the renal masses was 8.8 ± 4.6 cm (range: 1.3–24 cm), for clear cell carcinoma 9.2 ± 4 (range: 2–17cm), for papillary 8 ± 3.9 (range: 3–18 cm), for chromophobe 9.1± 4.6 cm (range: 2.5–20 cm), for AML 9.3 ± 8.3 (range: 4.5–24 cm), for XP 11.2 chromosomal translocation 11 ± 5 (range: 5.5–18 cm), and for oncocytoma 6.9 ± 2.5 cm (range: 3–10 cm).

There is no difference in the renal masses as regard their sizes and attenuation values at precontrast scan $p > 0.01$. For the relative postcontrast enhancement after correction of the attenuation values, there is a significant difference between the clear cell carcinoma and other different types, after exclusion of

AML, with sensitivity 84%, specificity 92%, and overall accuracy 93%, if we use 20 HU as a cutoff value between clear cell carcinoma and other renal masses.

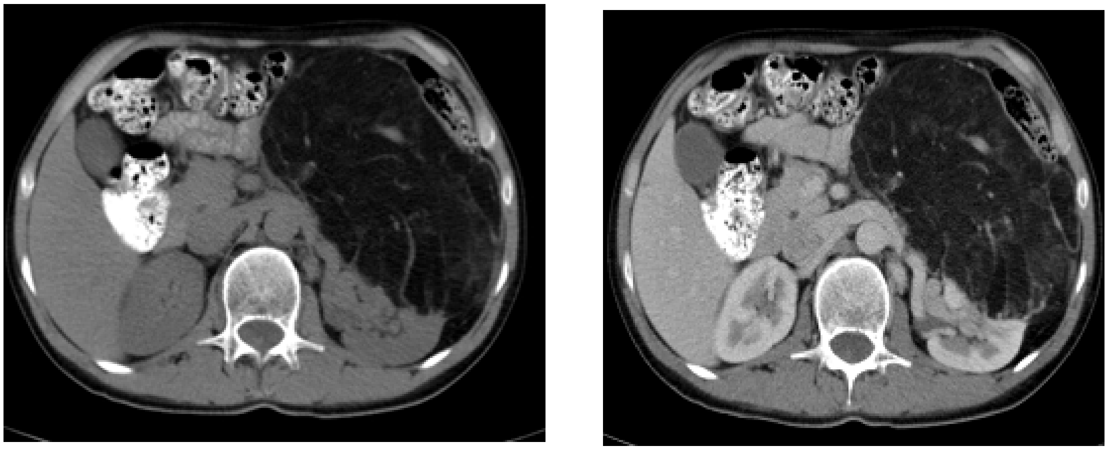

A B

**FIGURE 1.** A case of large left renal AML. (A) Nonenhanced axial CT scan shows large hypodense exophytic fatty tumor arising from the anterior aspect of the left kidney. (B) Postcontrast axial CT scan shows no enhancement by the fatty mass, with enhancing blood vessels inside.

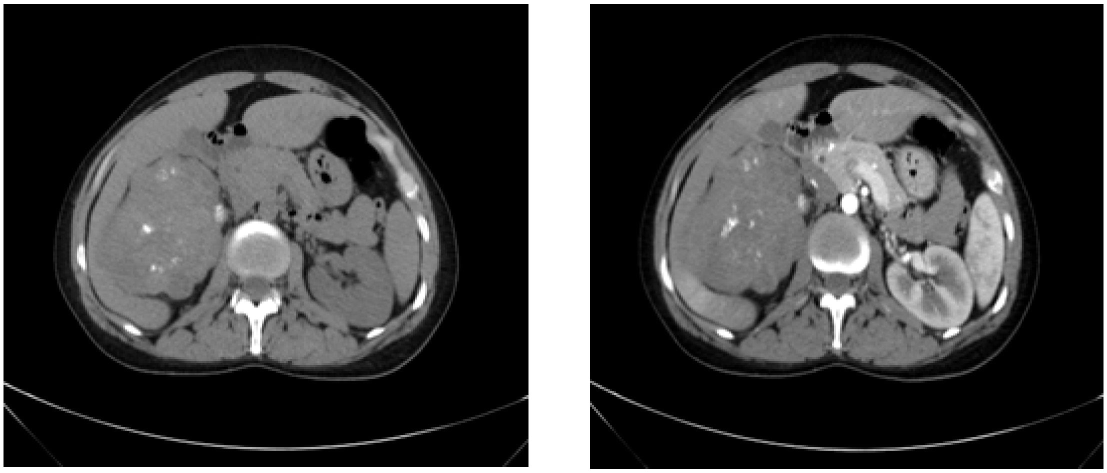

A B

**FIGURE 2.** A case of right RCC, papillary type. (A) Noncontrast axial CT scan of the abdomen shows a large soft tissue mass replacing the right kidney with foci of calcifications inside. (B) Axial CT scan of the abdomen at the CM phase shows mild enhancement by the mass.

## DISCUSSION

In the last decade, many authors investigated RCC and its subtypes using certain imaging features and correlated it with RCC subtypes[11,12,13,14,15]. Sheir et al.[12] and Kim et al.[15] used the morphological criteria as tumor size, calcification, and cystic degeneration, and they concluded that these criteria have a minor role in the differentiation between tumor subtypes. They also used the pattern of enhancement studied by Zhang et al.[11] and Herts et al.[14], and found that the most reproducible findings in differentiation between RCC subtypes was the degree of enhancement, as clear cell RCCs enhance to a greater degree than other subtypes. Although most of these studies included only malignant

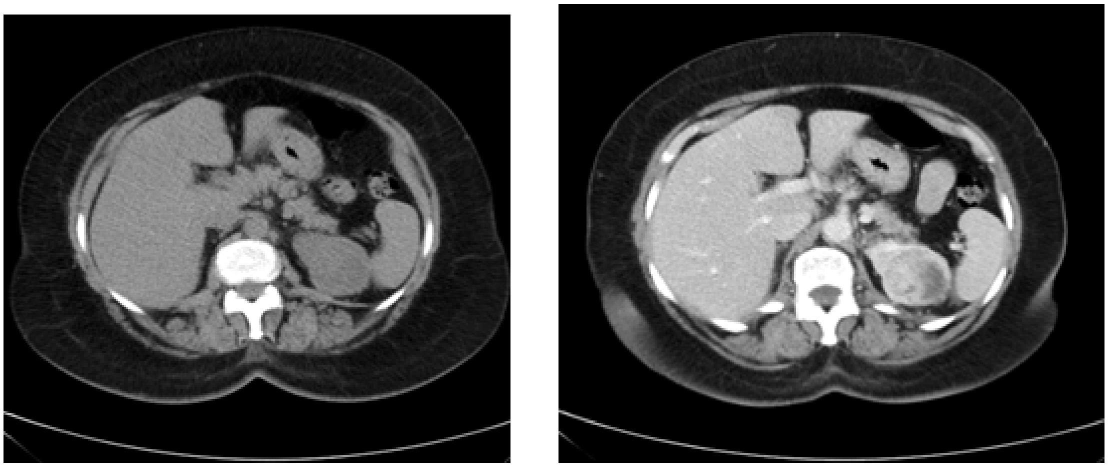

A B

**FIGURE 3.** A case of left RCC, clear cell type. (A) A nonenhanced axial CT scan of the abdomen shows upper polar posterior soft tissue mass isodense to the renal parenchyma. (B) Axial CT scan at the CM phase shows marked enhancement by the mass, with nonenhancing peripheral necrotic area.

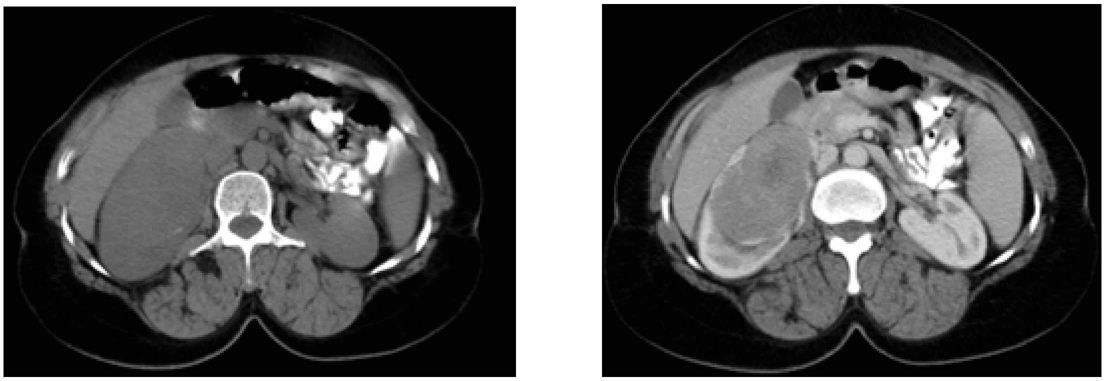

A B

**FIGURE 4.** A case of right RCC, chromophobe type. (A) Nonenhanced axial CT scan of the abdomen shows anterior midzonal soft tissue mass. (B) Postcontrast axial CT scan at CM phase shows moderately enhancing lesion with nonenhancing central area.

**TABLE 1**
**The Attenuation Values of Different Renal Mass Histologies**

| Histologies/Attenuation Values | Precontrast (HU); Mean ± SD (Range) | Postcontrast Corrected Attenuation Values; Mean ± SD (Range) |
|---|---|---|
| Clear cell RCC | 33.8 ± 8.8 (22–57) | 30.7 ± 11.8 (7–52) |
| Papillary RCC | 32.6 ± 11 (21–70) | –4.9 ± 10.1 (–15 to 8) |
| Chromophobe RCC | 31.9 ± 5.6 (22–44) | 1.8 ± 12.4 (–19 to 18) |
| XP 11.2 chromosomal translocation | 27.1 ± 3.4 (22–32) | 17.4 ± 16.2 (0–45) |
| Oncocytoma | 29.6 ± 9.1 (17–45) | 14 ± 17.3 (13–35) |

lesions or, in some cases, subgroups of malignant lesions in their analyses, a few studies included both malignant and benign tumors as did Zhang et al.[11] and Jinzaki et al.[13].

In a study by Kim et al.[15], the authors found that the degree of enhancement is the most useful parameter in differentiating subtypes of RCC, especially conventional renal carcinoma vs. nonconventional renal carcinomas with high validity ($p$ value < 0.05 in both the CM phase and the excretory phase). Conventional renal carcinoma showed stronger enhancement than nonconventional renal carcinomas in both the CM and excretory phases, and the tumors that enhanced more than approximately 84 HU in the CM phase and 44 HU in the excretory phase were likely to be conventional renal carcinoma. Although strong enhancement of conventional renal carcinoma has been observed in previous reports[17,18], the actual values of enhancement for differentiating conventional renal carcinoma from nonconventional renal carcinomas were reported by Kim et al. Some investigators believe that the strong enhancement of conventional renal carcinoma is caused by its rich vascular network and alveolar architecture at histological examination[15].

There are many factors that affect tissue enhancement. They may be intrinsic, such as patient's weight, cardiac function, state of hydration, and renal function, or extrinsic, such as amount and rate of injected contrast material[19,20]. Ruppert-Kohlmayr et al.[16] tried a new technique for correction of renal enhancement. The differentiation of renal clear cell carcinoma from renal papillary carcinoma, using the corrected attenuation in the CM phase, was accurate (95.7%). The nephrographic phase was also accurate (94.8%) in differentiating between renal clear cell carcinoma and renal papillary carcinoma.

Kim et al.[15] found lower cutoff values. These differences might be due to the inclusion of renal clear cell carcinoma, renal papillary carcinoma, and other lesions in their study, making no correction to the attenuation values.

In our study, we used a 64-slice MDCT scanner with thin-slice sections that allows easy characterization of the cases of AML, with detection of fatty areas noted, and it correlated well with the postbiopsy histopathology results with 100% accuracy. Application of our technique to correct the postenhancement attenuation values allows characterization of clear cell RCC from other RCC subtypes and the cases of oncocytoma with 89% sensitivity, 92% specificity, and 93% overall accuracy, and in our study, we reduced the radiation dose as no delayed scan was done for characterization of the masses.

## CONCLUSION

The use of a 64-slice MDCT scanner with application of enhancement values correction gives promising results. The criticism of our study is that we do correction of enhancement at the CM phase only and we also cannot separate the benign oncocytomas that need more workup in order to characterize them in the future.
